# Regulatory Effects of Caffeic Acid Phenethyl Ester on Neuroinflammation in Microglial Cells

**DOI:** 10.3390/ijms16035572

**Published:** 2015-03-11

**Authors:** Cheng-Fang Tsai, Yueh-Hsiung Kuo, Wei-Lan Yeh, Caren Yu-Ju Wu, Hsiao-Yun Lin, Sheng-Wei Lai, Yu-Shu Liu, Ling-Hsuan Wu, Jheng-Kun Lu, Dah-Yuu Lu

**Affiliations:** 1 Department of Biotechnology, Asia University, Taichung 413, Taiwan; E-Mails: tsaicf@asia.edu.tw (C.-F.T.); kuoyh@mail.cmu.edu.tw (Y.-H.K.); s123345142@gmail.com (J.-K.L.); 2 Department of Chinese Pharmaceutical Sciences and Chinese Medicine Resources, China Medical University, Taichung 404, Taiwan; 3 Department of Cell and Tissue Engineering, Changhua Christian Hospital, Changhua 500, Taiwan; E-Mail: ibizayeh0816@hotmail.com; 4 Graduate Institute of Basic Medical Science, College of Medicine, China Medical University, Taichung 404, Taiwan; E-Mails: carenyujuwu@hotmail.com (C.Y.-J.W.); wayson081024@yahoo.com.tw (S.-W.L.); yushuliu220@gmail.com (Y.-S.L.); lulu80620@yahoo.com.tw (L.-H.W.); 5 Graduate Institute of Neural and Cognitive Sciences, China Medical University, Taichung 404, Taiwan; E-Mail: lingirl831@hotmail.com; 6 Department of Photonics and Communication Engineering, Asia University, Taichung 413, Taiwan

**Keywords:** microglia, neuroinflammation, neurodegeneration, caffeicacid

## Abstract

Microglial activation has been widely demonstrated to mediate inflammatory processes that are crucial in several neurodegenerative disorders. Pharmaceuticals that can deliver direct inhibitory effects on microglia are therefore considered as a potential strategy to counter balance neurodegenerative progression. Caffeic acid phenethyl ester (CAPE), a natural phenol in honeybee propolis, is known to possess antioxidant, anti-inflammatory and anti-microbial properties. Accordingly, the current study intended to probe the effects of CAPE on microglia activation by using *in vitro* and *in vivo* models. Western blot and Griess reaction assay revealed CAPE significantly inhibited the expressions of inducible nitric oxide synthase (NOS), cyclooxygenase (COX)-2 and the production of nitric oxide (NO). Administration of CAPE resulted in increased expressions of hemeoxygenase (HO)-1and erythropoietin (EPO) in microglia. The phosphorylated adenosine monophosphate-activated protein kinase (AMPK)-α was further found to regulate the anti-inflammatory effects of caffeic acid. *In vivo* results from immunohistochemistry along with rotarod test also revealed the anti-neuroinflammatory effects of CAPE in microglia activation. The current study has evidenced several possible molecular determinants, AMPKα, EPO, and HO-1, in mediating anti-neuroinflammatory responses in microglial cells.

## 1. Introduction

Microglia, as sentinel cells in the brain, react to pathogens or diffusible mediators regulated by neighboring cells such as astrocytes or neurons [1,2]. Rapid microglial activation and associated inflammatory reactions are responses to combat the effect of insults, and contribute to immune-defense and tissue repair in the central nervous system (CNS) [3]. This acute activation is therefore considered to be protective [4]. By contrast, persistent microglial activation will ultimately results in vast production of proinflammatory mediators, chemokines and recruitment of peripheral immune cells [5] to the brain that characterizes chronic neurodegenerative diseases including multiple sclerosis, Parkinson’s disease, and Alzheimer’s disease [6,7,8]. Manifestations of activated microglia lies in its phenotypical alternation from a resting ramified state to active form marked by increased neurotoxic factors including the expression of inducible nitric oxide synthase (iNOS) and cyclooxygenase (COX)-2 [9,10]. Increasing evidence has revealed that iNOS mediates NO production [11], and induction of COX-2 in activated microglia causes the progressive neuronal damage [12] and brain tumor [13]. Therefore, pharmaceuticals that can deliver inhibitory effects on microglia are considered as a potential strategy to counter balance neurodegenerative progression [14].

Heme oxygenase (HO)-1, a stress-inducible protein, catalyzes a rate-limiting step of cellular oxidative agents against inflammatory responses or oxidative challenges [15]. Regulatory effects on inflammation and neuroprotection of HO-1 were also addressed in our previous study using microglia [16,17,18] and neuronal cells [19,20]. Other anti-inflammatory advances have shown signature neuroprotection of HO-1 via negative regulation of iNOS expression and iNOS-dependent NO production [21,22]. Moreover, induction of HO-1 has demonstrated protection from hypoxia-induced pulmonary hypertension by effectively modifying macrophage activation [23]. Erythropoietin (EPO) was originally recognized for its central role in erythropoiesis, which is crucial for the development of erythroid progenitors [24]. EPO has been identified as a neurotrophic factor and prevents neuronal apoptosis [25] by its ability to suppress tight junction openings and endothelial cell swelling [26]. Similarly beneficially, EPO was shown to ameliorate ischemic-induced microglial activation through TNF-α and IL-6 inhibition by up-regulation of HO-1 [27,28,29]. EPO maintains microglial integrity during oxygen-glucose deprivation [30]. The AMP-activated protein kinase (AMPK) in the brain and peripheral nervous system is an energy sensor that responds to ATP-depleting processes such as cellular stress or increases in the AMP/ATP ratio [31]. Accumulating evidence demonstrates that AMPK regulates cell migration, synaptic plasticity, and inflammatory responses [32]. Importantly, AMPKα is crucial for phagocytosis-induced macrophages from a pro-inflammatory to anti-inflammatory phenotype at the time of modulation of the inflammatory response [33]. The anti-inflammation of AMPKα on microglia has been addressed in our previous studies to be either activated by berberine or lycopene [16,34]. Induction of endogenous protective molecules in microglia may be useful as a therapeutic strategy in neuroinflammation and has benefits in neuroprotection.

Caffeic acid phenethyl ester (CAPE), as one of major bioactive ingredients of propolis, possesses unique biological activities including anti-oxidant, anti-inflammation, anti-tumor, and immune regulation [35,36,37]. As a natural food constituent, CAPE displays functional and structural similarity to polyphenol demonstrated to effectively activate AMPKα phosphorylation [38] that is beneficial over metabolic diseases, and cardiovascular diseases [39,40]. CAPE exerts strong anti-oxidant effects through blocking reactive oxygen species [41,42]. The anti-inflammatory activity of CAPE has been shown to inhibit arachidonic acid mediated COX-2 production [43], and NF-κB-dependent transcription. CAPE and its derivatives also have been reported to exert anti-oxidative and anti-inflammation properties in many cells [44,45]. Furthermore, several studies have suggested that CAPE holds a potential in neurodegenerative diseases [46]. It has been demonstrated that CAPE protects the integrity of the blood-brain barrier and protects against the loss of dopaminergic neurons in animal models [47,48]. In addition, CAPE was shown to exert direct neuronal protection through up-regulation of endogenous antioxidant and growth factor expression [35]. Our previous studies have addressed the effects of CAPE derivatives modulating inflammatory homeostasis [17].

Although the beneficial effects of CAPE in the nervous system have been investigated, detailed mechanisms of the regulation of microglia activation is not yet clear. In the present study, we addressed whether, in addition to inhibiting cytokine production, endogenous protective molecules also contribute to CAPE-regulated anti-inflammatory responses in microglial cells. Findings from the current study reveal that CAPE activates AMPKα phosphorylation concurrently with the up-regulation of EPO and HO-1 that may contribute to the microglial anti-inflammatory response.

## 2. Results

### 2.1. Caffeic Acid Phenethyl Ester (CAPE) Attenuates Microglial Activation Dose Dependently without Obvious Cytotoxicity

**Figure 1:**
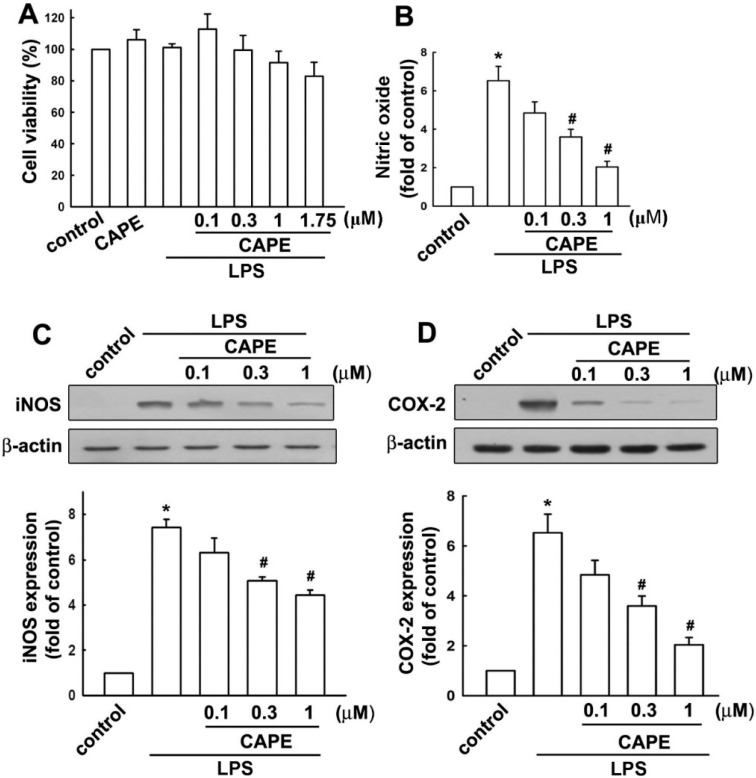
Caffeic acid phenethyl ester (CAPE) attenuates microglial activation without cytotoxicity. Treatment of CAPE (ranging from 0.1–1.75 μM) for 30 min prior to lipopolysaccharide (LPS) stimulation (200 ng/mL) for 24 h did not affect microglia viability as determined by 3-(4,5-cimethylthiazol-2-yl)-2,5-diphenyl tetrazolium bromide (MTT) assay (**A**); Treatment of CAPE effectively antagonized LPS-induced NO production as determined by Griess reagents (**B**); Treatment with various concentrations of CAPE for 30 min followed by stimulation with LPS. iNOS (**C**) and cyclooxygenase (COX)-2 (**D**) protein expression levels were determined by Western blot. Results of quantitative intensities were normalized to β-actin expressions. The results are expressed as means ± standard error (S.E.) from three independent experiments. * *p* < 0.05 compared with the vehicle control group; # *p* < 0.05 compared with the LPS treatment group.

**Figure 2:**
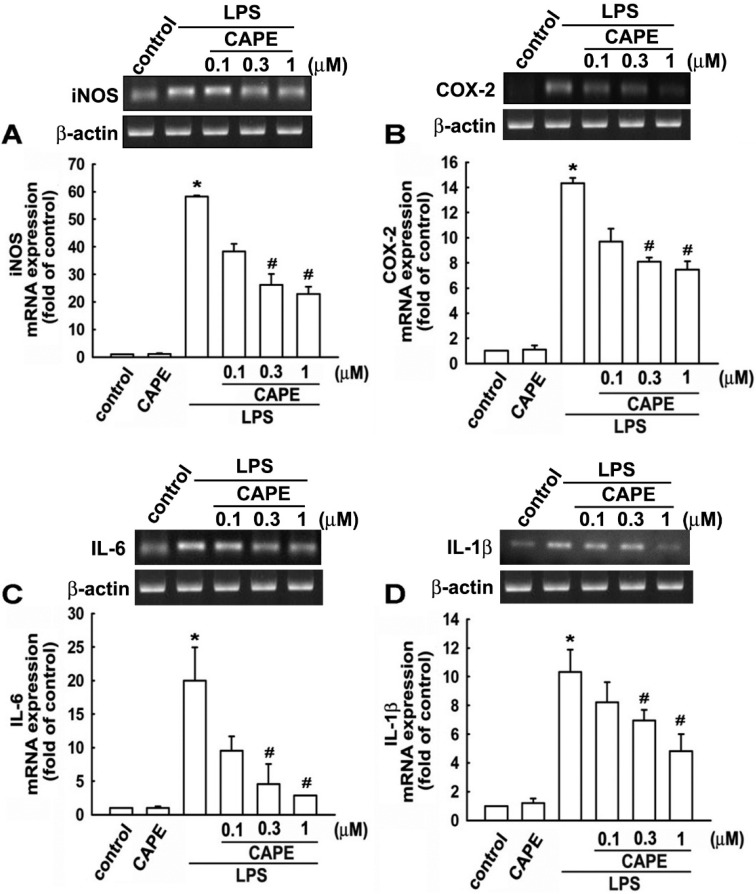
Inhibitory effect of CAPE on proinflammatory cytokine expression in microglia. Cells were pretreated with CAPE as indicated concentrations for 30 min followed by LPS stimulation (200 ng/mL) for 6 h. Relative mRNA levels of iNOS (**A**); COX-2 (**B**); IL-6 (**C**) and IL-1β (**D**) were determined by RT-PCR (**upper panel**). Quantitative results were determined by real time-PCR and GAPDH as an internal control (**lower panel**). The results are expressed as means ± S.E. from 3–4 independent experiments. * *p* < 0.05 compared with the vehicle control group; # *p* < 0.05 compared with the LPS treatment group.

Prior to study the effect of CAPE on microglia, the potential toxicity of CAPE was examined. Microglial cells were pretreated with CAPE (0.1 to 1.75 μM) for 30 min followed by lipopolysaccharide (LPS) treatment for another 24 h. The results showed that cell viability was not different from that of control cells following CAPE treatment (0.1 to 1.75 μM) as shown in Figure 1A. In addition, CAPE decreased nitric oxide production dose dependently after 24 h LPS stimulation (Figure 1B). To determine the effect of CAPE on proinflammatory mediator production, protein expressions of iNOS (Figure 1C) and COX-2 (Figure 1D) denoted microglial activation were examined, and found to display stepwise decrement in accordance with CAPE concentrations. To further evaluate the impact of CAPE on cytokine production following LPS stimulation, cells were treated with LPS in the presence or absence of CAPE, iNOS, COX-2, IL-6, and IL-1β, and gene expression was analyzed using RT-PCR and quantified with real time-PCR. CAPE significantly abolished LPS-induced iNOS (Figure 2A), COX-2 (Figure 2B), IL-6 (Figure 2C), and IL-1β (Figure 2D) production in a time-dependent manner.

**Figure 3:**
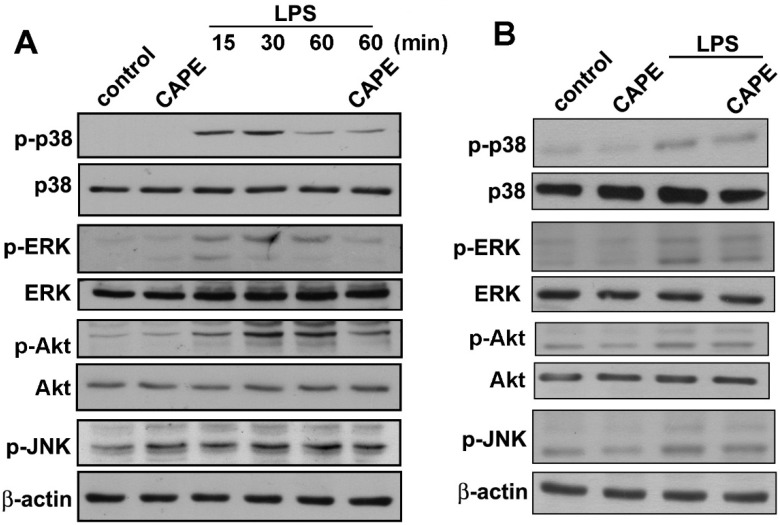
Inhibitory effects of CAPE on LPS-stimulated Mitogen-activated protein (MAP) kinase and Akt signaling pathways in microglia. (**A**) Following pretreatment with or without CAPE (0.3 μM) for 30 min, cells were treated with LPS (200 ng/mL) for 15, 30, or 60 min; and (**B**) Pretreatment with CAPE (0.3 μM) for 30 min followed by stimulation with LPS for 24 h. The levels of p-p38, p-ERK, p-Akt, and p-JNK were determined by Western blotting. CAPE significantly inhibited LPS-induced microglial p-p38, p-ERK, p-Akt and p-JNK expressions. Results expressed are representative of three independent experiments.

Next, we investigated the effect of CAPE on activity of p-p38, p-ERK, p-JNK, and p-Akt. Cells were treated with CAPE followed by LPS stimulation for another 15, 30, or 60 min. As shown in Figure 3A, p-ERK, p-JNK, and p-Akt but not p-p38 were reduced by CAPE treatment. Moreover, the effects of CAPE on kinase phosphorylation were also determined after LPS stimulation for 24 h. p-p38, p-ERK, p-JNK, and p-Akt displayed a drop upon CAPE treatment (Figure 3B). These results suggested that CAPE effectively inhibits inflammatory responses in microglial cells.

### 2.2. CAPE Induces Anti-Inflammatory and Protective Molecule Expressions in Microglial Cells

**Figure 4:**
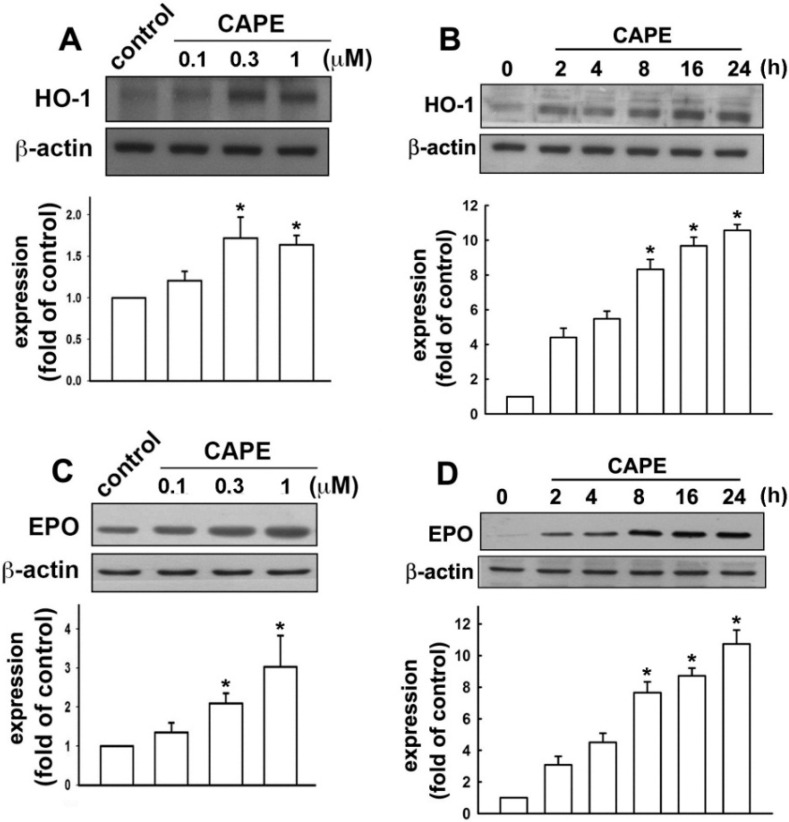
CAPE enhances the productions of anti-inflammatory molecules. Cells were treated with various concentrations of CAPE as indicated. Protein levels of hemeoxygenase (HO)-1 (**A**) and erythropoietin (EPO) (**C**) displayed up-regulation in response to CAPE concentrations. Single doses of CAPE treatment (0.3 μM) revealed a time-dependent increment (0, 2, 4, 8, 16, or 24 h) on the levels of HO-1 (**B**) and EPO (**D**). Results of quantitative intensities were normalized to β-actin expressions. Results were obtained from at least three independent experiments expressed as the mean ± S.E. * *p <* 0.05 as compared with the vehicle control group.

**Figure 5:**
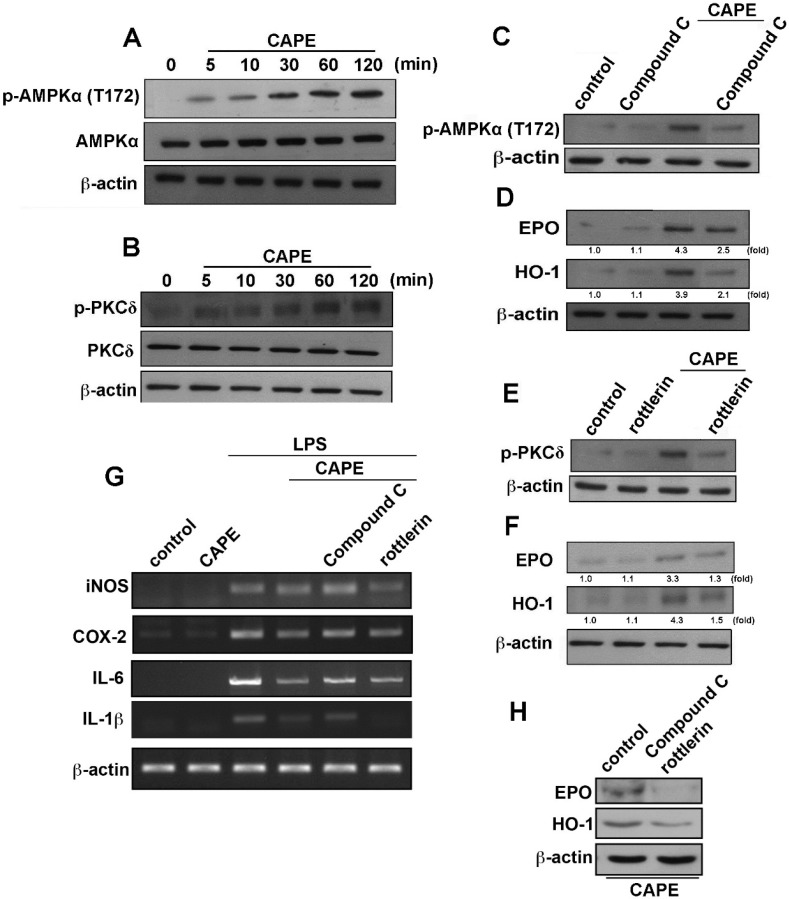
CAPE regulates the AMPKα and PKCδ signaling pathways. Treatment with CAPE for the indicated time periods, the phosphorylated AMPKα (**A**) and PKCδ (**B**) were determined by Western blot; Cells were pretreated with compound C (5 μM; **C**,**D**) or rottlerin (5 μM; **E**,**F**) for 30 min followed by stimulation with CAPE; Phosphorylated AMPKα and PKCδ were determined after CAPE stimulation for 60 min (**C**,**E**); EPO and HO-1 expressions were determined after CAPE stimulation for 24 h (**D**,**F**); Results of quantitative intensities were normalized to β-actin expressions; (**G**) Cells were pretreated with compound C or rottlerin for 30 min and added with CAPE for another 30 min before LPS treatment. mRNA levels of iNOS, COX-2, IL-6, and IL-1β were determined by RT-PCR; (**H**) Co-treatment with compound C and rottlerin followed by stimulation with CAPE for 24 h; EPO and HO-1 expressions were determined by western blot. Results expressed are representative of 3–4 independent experiments.

Cells were then treated with CAPE up to 24 h; HO-1 levels were surprisingly up-regulated in a dosage and time-dependent manner (Figure 4A,B). Moreover, CAPE also increased EPO protein expression time and dose dependently (Figure 4C,D). We further investigated the signaling pathway involved in anti-neuroinflammatory molecule expression of CAPE. Stimulation of cells with CAPE increased AMPKα phosphorylation at the Thr^172^ site (Figure 5A). On the other hand, CAPE also increased the phosphorylation of PKCδ within a transient period (Figure 5B). Treatment with AMPK antagonist compound C reduced AMPK activation (Figure 5C) and CAPE-induced EPO and HO-1 expression levels (Figure 5D). Similarly, rottlerin, commonly known as a PKCδ inhibitor, antagonized CAPE-induced PKCδ activation (Figure 5E). Treatment with rottlerin also down-regulated CAPE-induced EPO and HO-1 protein expression levels (Figure5F). Furthermore, treatment with compound C also effectively reversed the inhibitory effects of CAPE on proinflammatory cytokine expression (Figure 5G). However, we did not observe attenuation effects by treatment with rottlerin (Figure 5G). Moreover, co-treatment with compound C and rottlerin reduced CAPE-induced EPO and HO-1 expression levels (Figure 5H). These results demonstrated that CAPE mediates the expression of microglial anti-neuroinflammatory molecules through the AMPKα signaling pathway.

**Figure 6:**
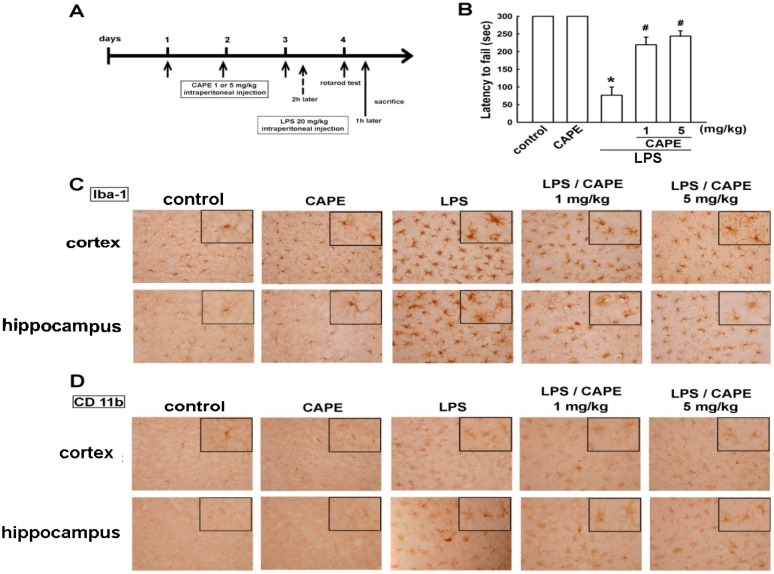
Effects of CAPE on LPS-induced microglial activation and motor dysfunction. The schematic representation shows the protocol for CAPE and LPS administrations (**A**); Mice were treated with CAPE (1 or 5 mg/kg) or vehicle once daily for 3 consecutive days. A single intraperitoneal injection of 20 mg/kg LPS was administered 2 h after the last CAPE injection. Motor coordination was analyzed by rotarod test. The improvement in gross motor function was observed in mice treated with CAPE compared to LPS-treated mice (**B**); CAPE-treated brain sections showed significant reduction of microglia activation in cortical and hippocampal regions identified with anti-Iba-1 (**C**) and CD 11b (**D**). Results were obtained from at least three independent experiments expressed as the mean ± S.E. * *p* < 0.05 as compared with the vehicle control group; # *p* < 0.05 as compared with the LPS treatment groups.

### 2.3. CAPE Ameliorates LPS-Induced Microglial Activation and Motor Incoordination

The *in vivo* study was carried out to determine the protective effects of CAPE on microglial activation and neuroprotection. CAPE was administrated to imprinting control region (ICR) mouse at a dose of 1 or 5 mg/kg for 3 consecutive days, and LPS was intraperitoneally injected 2 h after the last CAPE injection. Twenty-four hours after LPS injection, motor deficits and microglial activation were accessed using an accelerating rotarod test and immunohistochemical staining (protocol as shown in Figure 6A). Results revealed that LPS-treated mice displayed a reduced latency in the rotarod test indicating motor deficits as compared to the control group (Figure 6B). On the other hand, treatment with CAPE significantly ameliorated the motor impairments in LPS-injected mice (Figure 6B). Microglial activation was confirmed by immunohistochemical staining with the Iba-1 marker. Our previous studies have demonstrated that Iba1 is a useful marker for morphological change from the resting to ramified status of microglia in animal model [16,17,49,50]. Activated microglial cells displayed as thick, dense and fragmented processes with dark stained cell bodies, which were widely distributed in the cortical and hippocampal regions of the LPS-treated brain compared with the control (Figure 6C,D). However, microglial activation displayed stepwise decrements upon CAPE administration as shown by reducing bushy morphology (Figure 6C,D). We further used another important maker CD11b to confirm the microglial activation. As shown in our results, CAPE also slightly inhibited the morphology of CD11b immunoreactivity in the cortex and hippocampus. These results demonstrated that CAPE ameliorates microglial activation and motor incoordination in mouse model.

## 3. Discussion

The current study revealed that CAPE effectively inhibits expression of iNOS, COX-2, IL-6, and IL-1β in microglia. These results, along with previous studies, suggest that CAPE exerts protective processes by anti-inflammatory effects. Our study also demonstrated that CAPE is capable of up-regulating HO-1 and EPO, and we further described the regulatory mechanisms of HO-1 and EPO expression in microglia. Moreover, CAPE also exhibits suppressive effects against neuroinflammation induced by systematic LPS injection *in vivo*. The significant decrease of rotarod performance indicated motor incoordination, which was eliminated by CAPE treatment. In this study, we performed a complement of behavioral and histopathological examinations. Mice were systemically injected with LPS intraperitoneally, microglial activation was apparent, and globally distributed throughout the cortical and hippocampal regions. The reduction of microglial activation was maintained in those mice administered with CAPE. Gross motor function was in accordance with histological findings with surprising outcomes that CAPE significantly prevents the relapse of motor deficits. LPS injection induced hypertrophy of microglial cells, and Iba1-positive cells with highly branched processes in the cortex and hippocampus. Pretreatment with CAPE significantly inhibited LPS-induced microglia activation. We further used CD11b to confirm in immunohistochemical analysis that CAPE slightly inhibited microglia activation in the cortex and hippocampus. Although not sufficiently consistent, our previous studies indicatedthat Iba1 is a better marker for morphological change between the resting and ramified microglia. Thus, immunohistochemical results were consistent with rotarod performance indicating that CAPE suppressed microglial activation and possibly relative inflammatory responses that are attributable to improvement in motor control.

PKCδ is a novel serine/threonine kinase of the PKC family, and regulates several biological functions, such as cell proliferation, differentiation, migration, survival and apoptosis [51]. PKCδ has also been reported to play a key role in anti-inflammation and up-regulation of HO-1 [52]. Previous study also reported that AMPK is an upstream molecule that modulates PKCδ activation [53,54]. Furthermore, a notable induction of HO-1 expression via the PKC-δ pathway promotes resistance to oxidative stress [55].Our recent findings also reported that a synthesized CAPE derivative modulates neuroinflammation through PKCδ activation which regulates endogenous anti-inflammatory molecule SOCS3 expression in microglial cells [49]. Here, we observed that CAPE increased HO-1 and EPO expression via activation of the AMPKα and PKCδ pathways. However, administration with AMPKα inhibitor but not PKCδ inhibitor reversed the anti-inflammatory effects of CAPE (Figure 6G). The induction of protective molecules such as HO-1 and EPO expression and anti-inflammatory effects may be dependent experimental conditions. Further work is needed to elucidate the detailed regulatory mechanisms of PKCδ on CAPE modulation of anti-neuroinflammation and enhanced endogenous protective molecule expression in microglial cells.

Our previous results also demonstrated that AMPKα modulates the HO-1 molecule expression level [17,56]. Another report also showed that the AMPK pathway plays a pivotal role in cardioprotective effects of EPO [57]. The present study indicates that CAPE increases AMPKα phosphorylation and treatment with AMPK antagonist compound C reduced CAPE-induced EPO and HO-1 expressions. We provide *in vitro* and *in vivo* data in support for the anti-inflammatory effects of AMPKα on stimulation of CAPE in microglial cells. Current results clearly show CAPE is a potent anti-inflammatory mediator that eliminates LPS-induced iNOS, COX-2, IL-1β and IL-6 expressions in microglial cells. Treatment of CAPE significantly attenuates LPS-dependent MAP kinase and Akt signaling pathways. Inhibition of AMPK also attenuates HO-1 and EPO expression induced by CAPE. Importantly, our results also reveal that inhibition of AMPK dramatically reverses the inhibitory effects of CAPE on proinflammatory expression (Figure 5G). The current findings support previous reports that AMPKα plays a critical role on anti-inflammatory responses by CAPE. Regarding microglial activation, findings reported here reveal that induction of endogenous anti-inflammatory molecules HO-1 and EPO after microglia treatment with CAPE appears to be regulated by AMPKα. This result indicates that CAPE modifies its impact by balancing out the exaggeration carried by proinflammatory cytokines with up-regulation of anti-inflammatory mediators.

The results presented here suggest that AMPKα activation could perpetuate CAPE-mediated protective and anti-inflammatory effects in microglia, and offers new insights for developing therapeutic approaches to treat inflammatory-related disorders.

## 4. Experimental Section

### 4.1. Reagents and Antibodies

Primary antibodies against β-actin, ERK2, Akt, p38, p-ERK1/2, p-p38, p-AKT, p-JNK, and EPO were obtained from Santa Cruz Biotechnology (Santa Cruz, CA, USA). The antibody against ionized calcium binding adaptor molecule 1 (Iba 1), CD 11b was obtained from Wako Pure Chemical Industries (Osaka, Japan).Specific antibodies against HO-1, iNOS, and COX-2 were obtained from StressGen Biotechnologies (San Diego, CA, USA), BD Transduction Lab (Lexington, KY, USA), and Cayman Chemicals (Ann Arbor, MI, USA) respectively. The anti-p-AMPKα (phosphorylated at Thr^172^) and anti-p-PKCδ antibodies were obtained from Cell Signaling Technology (Danvers, MA, USA). HO-1 and PKCδ antagonists, compound C and rottlerin, were obtained from Calbiochem (San Diego, CA, USA) and ENZO Life Sciences (Farmingdale, NY, USA).

### 4.2. Cell Culture and Animal Experiments

The BV-2 murine microglial cell line was generated by infecting primary microglial cell cultures with a v-raf/v-myc oncogene carrying a retrovirus (J2). Since BV-2 cells retain most of the morphological, phenotypical, and functional properties described for freshly isolated microglial cells, they can be considered as immortalized active microglial cells. Cells were maintained in DMEM (Gibco, Grand Island, NY, USA) supplemented with 10% FBS in 5% CO_2_ and 95% humidified air environment at 37 °C.

Eight-week-old male ICR mice were purchased from the National Laboratory Animal Center (Taipei, Taiwan). Male mice were housed in groups of 5 to 6 under 12 h light-dark cycle (lights on at 08:00 hours) at constant room temperature with free access to food and water. All procedures for animal experiments were approved by the Animal Care Committee of China Medical University. Animals were manipulated in accordance with the Animal Care and Use Guidelines of the China Medical University (Taichung, Taiwan).

### 4.3. MTT Assay

The rate of cell growth was determined colorimetrically using the MTT assay [58]. Briefly, cells were incubated with the indicated concentrations of CAPE or 200 ng/mL of LPS alone, or pretreated with CAPE for 30 min prior to LPS treatment. The culture medium was discarded and the cells were incubated with a 0.5 mg/mL of 3-(4,5-dimethylthiazol-2-yl)-2,5-diphenyltetrazolium bromide (MTT, Sigma-Aldrich, St. Louis, MO, USA) solution for 2 h at 37 °C. The supernatants were removed and the formazan blue, which was formed in the cells, was dissolved with DMSO at room temperature for 5 min. Subsequently, absorbance was determined at 595 nm using a microplate reader (Thermo Scientific, Vantaa, Finland).

### 4.4. Western Blot Analysis

The cell lysates were prepared as described previously [59,60]. Briefly, cells were homogenized in lysis buffer for 30 min on ice. Thirty micrograms of protein was subjected to sodium dodecyl sulfate-polyacrylamide (SDS) gel electrophoresis. Proteins then were transferred to polyvinylidene fluoride (PVDF) membranes for 2 h and blocked with non-fat milk in phosphate-buffered saline (PBS). Specific proteins were probed with primary antibodies. Blots were visualized by enhanced chemiluminescence using Kodak X-OMAT LS film (Eastman Kodak, Rochester, NY, USA).

### 4.5. Nitrite Oxide Assay

Production of nitric oxide was assayed by measuring the nitrite levels of nitric oxide metabolism in the culture medium. Cells were seeded in 24-well culture plates (1 × 10^5^ cells/well) for 24 h. Thereafter, cells were treated with various specific inhibitors prior to stimulation with LPS (200 ng/mL). After 24 h stimulation, supernatants were collected. Nitrite production was determined by Griess reagent and measured at 550 nm using a microplate reader (Bio-Tek, Winooski, VT, USA).

### 4.6. Reverse Transcriptase (RT)-PCR and Quantitative Real-Time PCR (qPCR)

Total RNA was extracted with TRIzol reagent (Invitrogen, Carlsbad, CA, USA). The reverse transcription was performed using total RNA (2 μg) that was reverse-transcribed into cDNA and then amplified using oligonucleotide primers. Products were then separated, electrophoretically in an agarose gel and stained with ethidium bromide. Quantitative real-time PCR was performed according to our previous report [19,56]. Briefly, quantitative real-time PCR using SYBR Green Master Mix was performed with StepOne Plus System (Applied Biosystems, Foster City, CA, USA). The amplification cycles were carried out 40 cycles at 95 °C for 10 s and 60 °C for 1 min and analyzed with comparative *C*_t_ quantification. The threshold was set above the non-template control background and within the linear phase of target gene amplification to calculate the cycle number at which the transcript was detected (denoted as *C*_t_).

### 4.7. Immunohistochemistry

Microglia activation in the brain tissue was observed with immunohistochemistry according to our previous studies [37,61]. Mice were acclimated to their environment for 7 days before the experiments. All mice then received intraperitoneal injections of saline or 20 mg/kg LPS (*E. coli*, serotype 0127:B8) for 24 h. Mice were then deeply anesthetized and transcardially perfused with phosphate-buffered saline (PBS) containing 10% formaldehyde. Brains were post-fixed overnight in a 30% sucrose solution at 4 °C. Coronal sections of 30 μm thickness were sliced using a freezing sliding microtome cryostat (Leica CM305S; Leica, Rueil-Malmaison, France). Free-floating sections were quenched for 15 min in 1% hydrogen peroxide PBS, blocked with non-fat milk, and permeabilized with Triton X-100. The brain sections were incubated overnight at 4 °C with primary antibody against Iba-1 and CD 11b, as microglia markers. Following incubation with biotinylated secondary antibody, the sections were stained using the avidin-biotin complex (Vector Laboratories; Burlingame, CA, USA) and developed with diaminobenzidine (DAB; Vector Laboratories; Burlingame, CA, USA). Cortex and hippocampal Iba-1-positive microglia were digitally captured under a light microscope.

### 4.8. Rotarod Analysis

The rotard protocol was adapted as previously described in Hockly *et al.* [62]. Mice were placed on a smooth non-slip rod (UgoBasile7650, Linton Instruments, Diss, UK), which accelerated from 20 to 60 rpm over a period of 300 s. Data were calculated as the latency to fall in seconds.

### 4.9. Statistical Analyses

All experiments were performed at least three times in triplicates unless otherwise stated. Statistical analysis was performed using the software Graphpad Prism 4.01 (Graph Pad Software Inc., San Diego, CA, USA). Data are expressed as means ± S.E. Significant differences between the experimental and control groups were assessed by the Student’s *t*-test. The difference was determined to be significant if the *p* value was <0.05.

## 5. Conclusions

The results from the current study suggest that CAPE displays effectively anti-inflammatory activities and that its use may be a promising strategy in the treatment of neurodegenerative diseases.
